# Adenylate Cyclases of *Trypanosoma brucei*, Environmental Sensors and Controllers of Host Innate Immune Response

**DOI:** 10.3390/pathogens7020048

**Published:** 2018-04-25

**Authors:** Didier Salmon

**Affiliations:** Institute of Medical Biochemistry Leopoldo de Meis, Centro de Ciências e da Saúde, Federal University of Rio de Janeiro, Av. Brigadeiro Trompowski, Rio de Janeiro 21941-590, Brazil; salmon@bioqmed.ufrj.br; Tel.: +55-21-3938-6756

**Keywords:** *Trypanosoma brucei*, adenylate cyclase, cAMP signaling, innate immunity, inflammation, TNF-α

## Abstract

*Trypanosoma brucei*, etiological agent of Sleeping Sickness in Africa, is the prototype of African trypanosomes, protozoan extracellular flagellate parasites transmitted by saliva (*Salivaria*). In these parasites the molecular controls of the cell cycle and environmental sensing are elaborate and concentrated at the flagellum. Genomic analyses suggest that these parasites appear to differ considerably from the host in signaling mechanisms, with the exception of receptor-type adenylate cyclases (AC) that are topologically similar to receptor-type guanylate cyclase (GC) of higher eukaryotes but control a new class of cAMP targets of unknown function, the cAMP response proteins (CARPs), rather than the classical protein kinase A cAMP effector (PKA). *T. brucei* possesses a large polymorphic family of ACs, mainly associated with the flagellar membrane, and these are involved in inhibition of the innate immune response of the host prior to the massive release of immunomodulatory factors at the first peak of parasitemia. Recent evidence suggests that in *T. brucei* several insect-specific AC isoforms are involved in social motility, whereas only a few AC isoforms are involved in cytokinesis control of bloodstream forms, attesting that a complex signaling pathway is required for environmental sensing. In this review, after a general update on cAMP signaling pathway and the multiple roles of cAMP, I summarize the existing knowledge of the mechanisms by which pathogenic microorganisms modulate cAMP levels to escape immune defense.

## 1. Introduction

**Figure 1:**
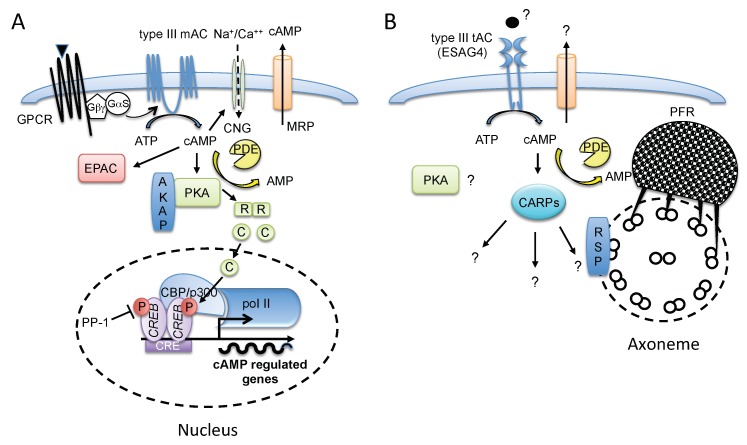
Schematic representation of cAMP signaling in mammalian cells (**A**) vs. *T. brucei* (**B**), highlighting the contrast between canonical mammalian cAMP/PKA signaling pathway and the African trypanosomes’ cAMP signaling, which mainly concentrates in the flagellum and is characterized by the almost total absence of polII transcriptional regulation. (**A**) In mammals, ligand (triangle) binding activates GPCR, which undergoes a conformational change and then activates the G proteins by promoting the exchange of GDP/GTP associated with the Gα subunit, triggering its dissociation from the Gβ/Gγ dimer to activate type III AC. AC produces cAMP from ATP. High local levels of cytosolic cAMP lead to activation of PKA holoenzyme, which binds the AKAP through a hydrophobic dimerization domain of the PKA-R subunit, Epac or CNG channel. Upon cAMP binding to PKA-R, PKA-C subunits dissociate, then translocate to the cell nucleus, and induce the phosphorylation of transcription factors, such as CREB, to activate cAMP-driven genes. CREB inactivation is promoted by a phosphatase (e.g., PP-1). PDE and MRP decrease intracellular cAMP levels and counterbalance the intracellular cAMP effect. (**B**) In *T. brucei*, a putative ligand (circle) or membrane stress (hypotonic, acidic, proteolytic) activates flagellar type III AC (prototype ESAG4 in bloodstream form). This AC is topologically similar to receptor-type GC and produces cAMP from ATP. In *T. brucei* no classical PKA effector is activated by cAMP; instead, the cAMP targets are CARPs, components of unknown function, which participate in a putative novel cAMP signaling pathway. RSP represents an AKAP-like protein linked to the flagellar axoneme (RSP3/AKAP97-like); PFR represents the parafagellar rod structure of the flagellum, which is linked to PDEs (TbPDEB1/B2). No CNG channels or Epac have been characterized in trypanosomatids, and there is no evidence for cAMP secretion via membrane channels. *AKAP*, *A*-kinase anchoring protein; CBP, cAMP-binding protein; CARP, cAMP response protein; CNG, cyclic nucleotide-gated ion channel; CRE, cAMP-response elements; CREB, cAMP response element-binding protein; EPAC, exchange protein directly activated by cAMP; Gαs, stimulatory G protein alpha subunit; Gβγ, G protein beta gamma subunits; GPCR, G-protein-coupled receptor; MRP, multidrug resistance protein; PDE, phosphodiesterase; PFR, paraflagellar rod; PKA, protein kinase A; PolII, RNA polymerase II; RSP, radial spoke protein.

Cyclic AMP (cAMP) is a ubiquitous metabolite produced from ATP by adenylate cyclase (AC). This molecule is involved in regulation of enzyme activities and/or gene expression in all organisms except in bacteria of the Firmicutes group [1]. Although this paradigmatic signaling molecule is involved in numerous and varied physiological processes ranging from carbon catabolite repression in bacteria [2] to chemotaxis mediation in *Dictyostelium* [3] and the action of hormones in superior eukaryotes, the downstream effectors of the cAMP pathway and their biological functions still represent an open issue that needs to be clarified [4]. In 1957, Earl Sutherland and his collaborators reported the presence of a heat-stable «active factor» (HSF), later proved to be cAMP, which was induced by glucagon or epinephrine, and which stimulated the activity of glycogen phosphorylase in cell-free homogenates of dog liver [5]. With these findings, a new concept of second messenger emerged, initiating the study of intracellular signaling pathways [6]. Years later, the cascade of AC activation in response to a great number of extracellular ligands such as hormones or autacoids (prostaglandins E2 and I2, histamine, serotonin) was shown to include the AC-stimulating G protein subunit (Gαs) of a G-protein-coupled receptor (GPCR) [7,8], and to culminate in triggering a wide range of cellular responses through activation of a serine/threonine protein kinase A (PKA) [9,10], exchange proteins directly activated by cAMP (Epacs) [11,12], cyclic nucleotide-gated (CNG) channels [13], and finally cyclic nucleotide phosphodiesterases (PDEs), crucial enzymes that hydrolyze cAMP to 5′-AMP [14,15] (Figure 1A).

In many organisms, the intracellular concentration of cAMP not only depends on the rate of synthesis and hydrolysis of cAMP, but also on its secretion to the medium via specific transport systems. In mammalian cells, for example, cAMP efflux is mediated by multidrug resistance protein (MRP), which also functions as an extracellular controller of the GsPCR-induced cell response [16]. Furthermore, cAMP-dependent regulatory pathways exhibit great variation depending on the activation of multiple AC isoforms. Indeed, ACs form a large and diverse family divided into four different main classes of phylogenetically independent origins (phylogenetic convergence) [17]. Whereas classes I and II, are exclusive to bacteria and class IV is found in archaebacteria as well as in bacteria, class III is universal. The latter characterizes the mammalian ACs, which can be divided into two types: the membrane-bound ACs represented by nine isoforms (type I to type IX, [18,19]) and a soluble AC (sAC) [20]. Similarly, PDEs occur in multiple isoforms (more than 40 isoforms divided into 11 different families [21]) often with distinct subcellular locations. Typically, in mammalian cells, a GPCR (except for the sAC) transmits a specific signal by creating a cAMP gradient within a specific location within a particular type of cell. Signal integration is carried out by a few PKAs, PDEs and phosphatases anchored to specific sites in the cell by scaffold proteins, as the A-kinase anchoring proteins (AKAPs) [22]. AKAPs are targeted near specific substrates such as PKA for local activation or PDEs for signal termination. Because of its high diffusion constant (~500 μ^2^ s^−1^) [23] and the relatively low abundance of the cAMP binding sites, an accurate spatio-temporal control through the action of PDEs is essential to fine-tune both the amplitude and duration of the cAMP signal, as required when it is necessary to activate a subset of PKA molecules [24] or cyclic nucleotide-gated channels [25].

Typically, upon cAMP binding to regulatory subunits of PKA in mammals, C subunits dissociate and phosphorylate numerous cytosolic (metabolic enzymes) or nuclear proteins after nuclear translocation [26] (Figure 1A). Regulation of transcription by PKA is mainly achieved by direct phosphorylation of cAMP-responsive bZIP transcription factors, including cAMP-response element binding protein (CREB), members of the cAMP-responsive element modulator/inducible cAMP early repressor (CREM/ICER) protein family [27], activating transcription factor-1 (ATF-1), and also nuclear receptors. Phosphorylated CREB, CREM, and ATF-1 interact with the transcriptional coactivator CREB-binding protein (CBP) or its paralogue p300 bound to cAMP-response elements (CREs) in target genes to mediate transcriptional activation through association with RNA polymerase II (Pol II) complexes and through intrinsic histone acetyltransferase activity [28]. Activated target genes regulate diverse cellular responses, including proliferation, survival, and differentiation. Target gene inactivation is carried out by the serine/threonine phosphatases PP-1 [29] and PP2A [30], which mediate dephosphorylation of CREB.

As discussed later in this review, the canonical mammalian cAMP/PKA signaling highly contrasts with that of flagellate African trypanosomes, in which an elaborate novel cAMP signaling pathway concentrates in a highly specialized organelle, the flagellum, critically involved in motility, cell division and morphogenesis, immune evasion, and sensory perception (Figure 1B). Trypanosomes appear to have lost the ability to regulate transcription mainly by RNA polymerase II, and gene expression relies mainly on post-transcriptional regulations in response to environmental changes [31,32]. Therefore, the pivotal role of the PKA-like kinase in gene expression regulation is not apparent. An RNAi screen for proteins associated with the AMP/cAMP response revealed the key actors in gene expression within a cohort of genes that encode proteins involved in purine metabolism and signal transduction. Among them are several gene regulators, including several RNA-binding proteins (e.g., RBP7, critically involved in the slender to stumpy differentiation of the pleomorphic parasites occurring in the bloodstream of the host) [33]. A PKA homologue and its regulatory subunits are present in all three genera of trypanosomatids, and its activity has been reported to be positively correlated with cAMP levels produced by the oxygen-dependent, globin-coupled, heme-containing AC from *Leishmania major* [34]. In *T. brucei*, however, PKA-like kinase activity is stimulated not by cAMP but by high concentrations of cGMP (*K*_d_ in the μM range) whose presence has still not been confirmed in these parasites [35]. Moreover, in trypanosomes no classical cAMP effectors such as CNG channels or Epac have been characterized and there is no evidence for cAMP secretion via membrane channels. Conversely, a novel PKA-independent cAMP pathway involving several cAMP response proteins (CARPs) of unknown function, some of which are kinetoplastid-specific, was recently characterized in both *T. brucei* and *T. cruzi* [36,37].

## 2. Role of cAMP in Innate and Adaptive Immunity and Pathogen Strategies to Counteract Immunity

Among its multiple roles in cell physiology, cAMP regulates pro- and anti-inflammatory activities [38]. Typically, signaling cascades that trigger increases in intracellular cAMP, thereby promoting CREB phosphorylation, also serve to lower the synthesis of pro-inflammatory mediators (cytokines such as TNF-α [39] or IL-12 [40] and chemokines such as CCL3-4 [39] and the pro-inflammatory lipid mediator leukotriene B_4_ [41]), and increase the production of anti-inflammatory factors such as IL-10 [39]. It was hypothesized that CREB directly inhibits NF-κB activation by blocking the binding of CBP to the NF-κB complex [42]. In mice, increased cAMP levels in general appear to decrease monocyte inflammatory functions (classical M1-type cells, Ly6C^high^) [43]. This induces the expression of an orphan nuclear receptor Nr4a1 (Nur77) [44] that down-regulates the expression of pro-inflammatory genes towards a reparatory monocyte phenotype (that is, resolution-phase M2-type macrophages but expressing M1 markers [45]). In other myeloid cells such as dendritic cells (DCs), cAMP inhibits the release of pro-inflammatory mediators (TNF-α, IL-17, IFN-γ) [46] and induces the release of anti-inflammatory mediators, such as IL-10 [47]. In addition, cAMP limits B and T cell activation through B cell and T cell receptors [43]. Increased levels of intracellular cAMP in T cells strongly impairs both IL-2 production and T cell proliferation, which suggests that cAMP is an essential component of the suppressive mechanism in T cells [48,49]. Remarkably, regulatory T cells (Treg) contain high amounts of intracellular cAMP, which is directly injected into effector T cells via gap junction intercellular communication, leading to their suppression in order to maintain the balance of the immune tolerance [43,50].

Conversely, infection of cells by microorganisms activates the pro-inflammatory response. The initial sensing of infection is mediated by innate pattern recognition receptors (PRRs), which recognize components of bacteria, fungi, protozoans, and viruses called *Pathogen-Associated Molecular Patterns (PAMPs).* These PRRs include Toll-like receptors (TLR 1-9 in human and mice), retinoid acid-inducible gene I (RIG-I)-like receptors (RLRs), nucleotide-binding oligomerization domain (NOD)-like receptors (NLRs) and C-type lectin receptors [51,52]. TLRs, RLRs and several NLRs activate intracellular signaling pathways that converge on activation of NF-κB and mitogen-activated protein kinase (MAPK) leading to transcriptional expression of pro-inflammatory mediators (pro-inflammatory cytokines, type I interferons (IFNs), chemokines, adhesion molecules and antimicrobial peptides) and proteins involved in the modulation of PRR signaling that promote innate immune responses.

TLRs (and other pro-inflammatory signals such as IL-1β and TNF-α) are the classical activators of the NF-κB pathway, which activates TLR adapter molecules MyD88 and TRIF and posterior phosphorylation and degradation of IκB (NF-κB inhibitor), allowing the active NF-κB transcription factors RelA (p65)/p50, which form the NF-κB heterodimeric complex to enter the nucleus, resulting in NF-κB pathway activation [42]. An important aspect of the regulation of the transcriptional activity of NF-κB complex is the phosphorylation of its transcription factor p65 by PKA in a cAMP-independent manner. The degradation of IκB, which was previously maintained in an inactive state through association with IκB-α, results in activation of the catalytic subunit of PKA, promoting the phosphorylation of p65 subunit and concomitant activation of NF-κB [53,54]. However, optimal transcriptional activity of NF-κB requires interaction of the RelA (p65) subunit with CBP or p300. Activation of CREB by PKA leads to CREB binding to the same region as CBP/p300, competing with the RelA component of NF-κB and thus inhibiting NF-κB [55,56]. However, the significance of this hypothetical competition between CREB and CBP/p300 in a physiologic setting is unclear [42].

**Figure 2:**
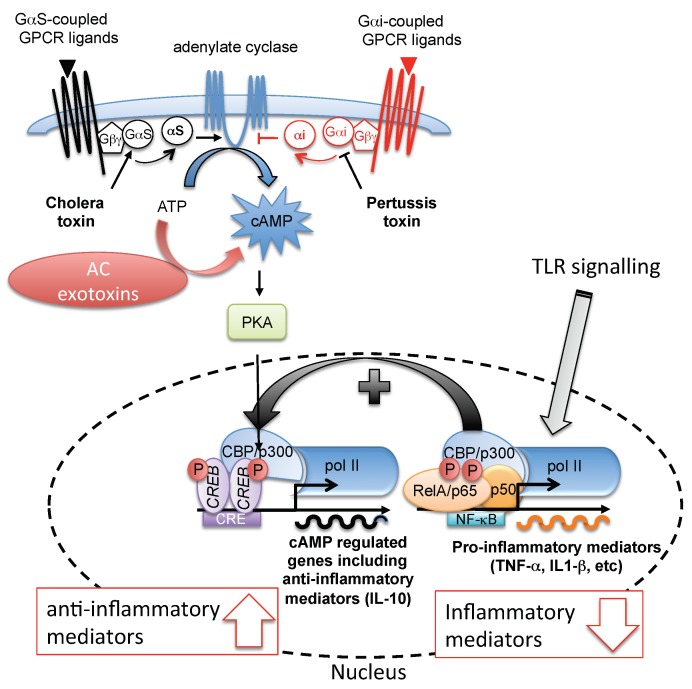
Pathogen strategies to counteract immunity by subversion of host cell cAMP signaling. Several extracellular bacterial pathogens possess virulence effectors, which increase cAMP levels in host cells, either by a G-protein modifying ADP ribosylation (pertussis toxin and cholera toxin) or by secreted AC exotoxins. Normally, the binding of an agonist (triangle) activates GPCR, which undergoes a conformational change leading to liberation of either the Gαs or Gαi subunit from the Gβγ subunit complex to, respectively, activate or inhibit the production of cAMP by AC. Pertussis toxin and cholera toxin produced by some pathogenic bacteria cause elevated cAMP levels through ADP-ribosylation of either the Gαi subunit to prevent AC inhibition or of the Gαs subunit to constitutively activate AC, respectively. TLRs binding of components of pathogenic bacteria (PAMPs) by TLRs triggers activation of NF-κB (RelA (p65)/p50) leading to transcriptional expression of pro-inflammatory mediators. Conversely, the production of high cellular cAMP levels by exotoxins increases the activation of CREB through PKA (driven anti-inflammatory mediator), which then competes with p65 for limiting amounts of CBP, resulting in fewer p65/CBP complexes, which are required for NF-κB activities that drive TNF-α expression (curved black arrow). CBP, cAMP-binding protein; CRE, cAMP-response elements; CREB, cAMP response element-binding protein; Gαi, inhibitory G protein alpha subunit; Gαs, stimulatory G protein alpha subunit; Gβγ, G protein beta gamma subunits; GPCR, G-protein-coupled receptor; NF-κB, nuclear factor-κB; PolII, RNA polymerase II.; RelA, Rel-associated protein; TLR, Toll-like receptor.

The CREB regulation of NF-kB signaling, master regulator of the inflammatory response, explains why several pathogenic microorganisms such as trypanosomatids have evolved mechanisms to exploit parasite and/or host cell cAMP signaling as a virulence factor. These include coordination of intracellular processes leading to virulence gene expression that is triggered by extracellular signals from the host environment, and manipulation of host immunity by directly or indirectly increasing cAMP levels in host cells during infection [38] (Figure 2).

Several bacteria directly manipulate the cAMP intracellular levels by introducing exogenous microbial ACs [57] (*Bordetella pertussis*, *Bacilus anthracis* [58], *Mycobacterium tuberculosis* [59], *Pseudomonas aeruginosa* [60], *Yersinia pestis* [61]) or by intoxicating the host cell with preformed cAMP or exotoxins [59,62] such as cholera toxin (CT) of *Vibrio cholerae*, pertussis toxin (PT) of *B. pertussis* or labile toxin (LT) of *E. coli*. These latter toxins modulate the activity of the endogenous host ACs by altering the function of heterotrimeric G proteins (ADP-ribosylation of Gαi, Gαs) increasing AC activity [63]. All these toxins contribute by elevating cAMP levels to suppress innate immune functions by modulating inflammatory mediator expression. As a result, the phagocytic response is reduced and intracellular killing of ingested pathogens is also attenuated [38].

**Figure 3:**
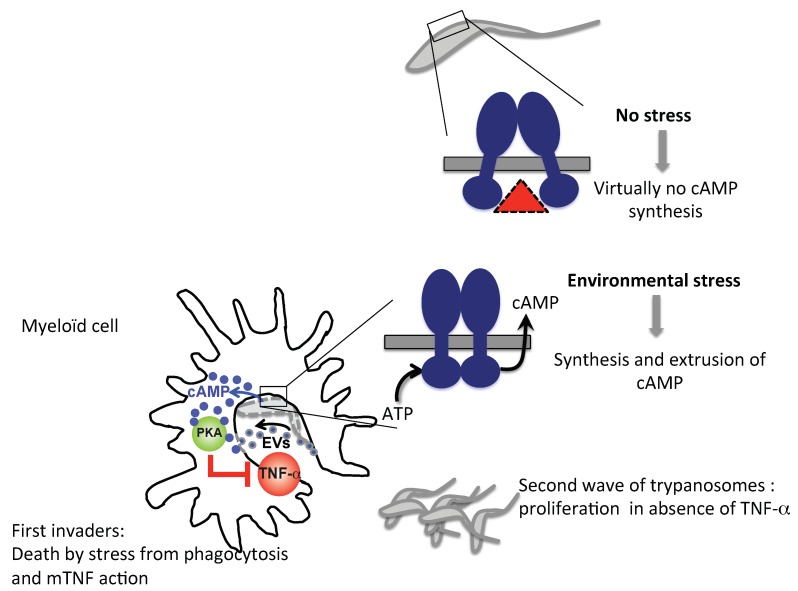
Proposed model to explain how the sacrifice of some parasites is thought to disable the innate immune response mediated by myeloid cells, allowing efficient host colonization by a second wave of invaders. In the absence of membrane stress, low basal levels of intracellular cAMP are produced following the combined actions of TbPDEB1/2 and low dimerization tendency of AC catalytic domains (red triangle). In the presence of stress, upon phagocytosis of the parasites by the acidic phagosome environment or following mTNF-mediated trypanolysis, CHDs dimerize, triggering a massive synthesis of cAMP that is translocated through an unknown mechanism into the myeloid cells cytosol or by phagocytosis/direct membrane fusion of extracellular vesicles (EVs) produced during phagocytic stress, blocking the synthesis of mTNF through activation of the host PKA. In this altruistic strategy, the sacrifice of the first pathogen invaders enables a second wave of trypanosomes to proliferate in the absence of local TNF-α, essential for the establishment of infection.

Differently from bacterial AC toxins, *T. brucei* possesses on its surface, in particular on its flagellum [64], a battery of around 80 AC isoforms [65,66], which seem to function collectively as a tolerogenic tool by producing large amounts of cAMP during phagocytosis by myeloid cells at the beginning of the innate immune response (Figure 3).

The overproduction of cAMP (~250-fold above the basal cellular content, which is around 0.67 μM [66]) inhibits the synthesis of trypanolytic TNF-α [67,68] by liver M1-type Ly6C^high^ inflammatory monocytes, inflammatory DC and macrophages, through activation of the host PKA [66]. These observations have led to the development of a model to explain how African trypanosomes succeed in disabling the innate immune response mediated by myeloid cells, thereby allowing the parasite to efficiently colonize the host (Figure 3). In this altruistic strategy, wherein the sacrifice of some individuals promotes the survival of others, the stress induced by phagocytosis of kamikaze parasites by M1-type myeloid cells leads to a disabling of the M1-mediated innate immune response required for parasite control, and enables the initiation of the first wave of parasitemia, essential for the establishment of chronic infection [66]. In the next chapter, we discuss in depth the mechanism by which the parasite hijacks the host cAMP pathway. This mechanism differs from that of bacterial toxin cyclases, which harbor completely different architectures involved in the mechanism of AC translocation [69].

## 3. *T. brucei* cAMP Signaling Pathway: From an Environmental Sensing Mechanism to an Innate Immune Evasion System

### 3.1. Receptor-Type ACs, a Hallmark of Trypanosomatids

**Figure 4:**
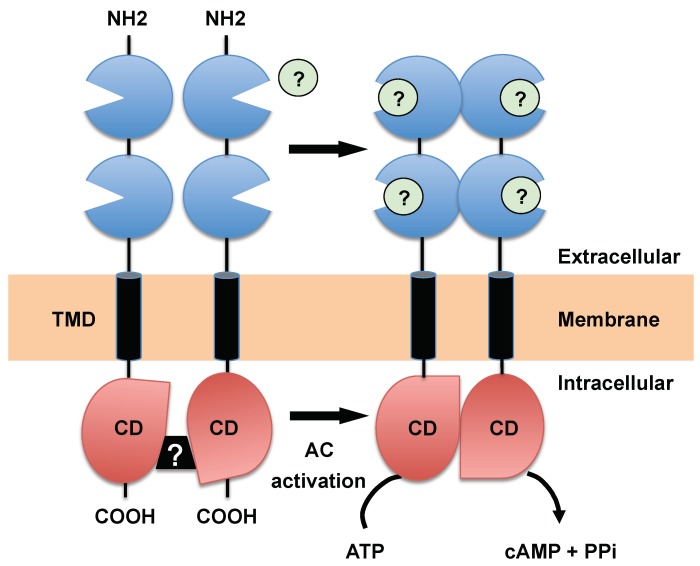
Predicted topology and hypothetical mode of activation of the receptor-type AC from *T. brucei.* A single transmembrane domain (TMD) separates an extracellular N-terminal domain containing two Venus flytrap (VFT) domains from a catalytic homology domain (CHD). AC activation is triggered by efficient head-to-tail dimerization of the CHDs that was postulated to be mediated through the N-terminal domains. This would involve conformational changes (e.g., dimerization) of VFT domains upon ligand binding (green circle) or membrane stress. Black box with question mark represents the allosteric inhibition mediated through the Δ-subdomain/phosphorylation. Membrane-bound forms appear to form homodimers and multimeric complexes [94].

Although the basic pathway that represents the classic view presented in all biochemistry textbooks culminates in the activation of the most common downstream effector (i.e., PKA), signaling pathways controlled by cAMP can vary greatly between tissues within a specific organism and across organisms. In this respect the trypanosomatids, protozoan parasites belonging to the order Kinetoplastida, are surely among the most intriguing organisms, with signaling pathways that are very different from those of their mammalian hosts. In these parasites the molecular controls of the cell cycle and environmental sensing are elaborate and concentrated at the flagellum, which is involved in motility [70], morphogenesis [71] and cytokinesis [72]. Genomic analyses suggest that these parasites differ considerably from the host in signaling mechanisms, lacking typical signaling receptors (e.g., receptor-linked tyrosine [73]), heterotrimeric G protein, as well as SRC homology regions 2 and 3 (SH2, SH3 domains), phosphotyrosine-binding (PTB) domain [74], receptor-type GC [74] and transcription factors [75], although they do have the receptor-type ACs that are topologically similar to GC-coupled receptors of higher eukaryotes [76] and belong to a sub-family of class III AC [17]. ACs of class III represent the archetypal AC present in all kingdoms of life, which characterizes unicellular eukaryotes and higher eukaryotes. Because of the divergent nature of stimuli, which have an impact on these enzymes, highly individualized class III ACs have evolved using different architectures and mode of enzymatic regulation [77]. Most of them are multi-modular proteins and possess one or two catalytic domains, termed CHD (catalytic homology domain of the mononucleotidyl cyclases) [78,79]. These domains contain a central ferredoxin-like βαββαβ structural motif, conserved in several other enzymes that catalyze the nucleophilic attack of a 3′-hydroxyl upon a 5′ nucleotide phosphate, as in the palm domain of type I DNA polymerase. The catalytic core consists of a central five-stranded, antiparallel β-sheet (β2-β3-β1-β4-β5), with three α-helices (α1–α3) on the back face of this sheet and another helix (α4) on the front face [78,79,80]. The two CHDs form a head-to-tail dimer and generate the active form [79], as do the mammalian 12-transmembrane ACs, which upon activation form a pseudo-heterodimer C1 and C2. C1 and C2 domains are responsible for forskolin- and G-protein-stimulated catalysis, the C1 domain providing residues that contribute to metal-binding (Mg^++^, Mn^++^) [81] while the C2 domain contains residues that confer nucleotide substrate specificity [78]. Trypanosomes ACs possess an activation mechanism very similar to that of receptor-type GCs, in which the CHDs upon activation transiently form a homodimer [82] (Figure 4).

High-resolution structures of the CHD of two trypanosomal ACs (tACs) (GRESAG4.1 and GRESAG4.3) indicates that these enzymes are structurally highly related to the class III ACs and follow an almost identical catalytic mechanism [82], although the tACs are not activated by the diterpene forskolin [83] nor significantly inhibited by P-site inhibitors, 2′-deoxy-adenosine and its 3′-mono- or polyphosphate derivative such as pyrophosphate [82]. P-site inhibitors act as dead-end inhibitors of product release (PPi), stabilizing an enzyme-product (E-PPi) complex by binding at the active site [84]. This relative insensitivity towards P-site inhibitors as already described for bacterial soluble AC from *B. pertussis* [85] may be due to a looser association between AC monomers and not to structural differences in the active site [82]. While calcium was reported to stimulate AC activity (two- to eightfold) of the bloodstream form (an effect attributed to the bloodstream-specific AC ESAG4) [86,87], no such stimulation was detected either for the CHD domain of either ESAG4, GRESAG4.1 or GRESAG4.3 [82]. Interestingly, the regulatory region corresponding to the α3-β4 loop of the C2 domain of mammalian class III AC contained in the case of tAC an extra 36 amino-acid sequence forming two additional α-helices (α3A and α3B) [82]. This region, which is conserved in trypanosomatids, forms a deltoid structure, termed Δ-subdomain, which encloses a small internal cavity binding stereospecifically a single D-DTT molecule. This subdomain is thought to correspond to an allosteric regulator site [82]. Regarding the overall topology, all AC isoforms of trypanosomatids are built up similarly to the mammalian receptor-type GC and possess an ample variable extracellular domain (of around 90 kDa) separated from the cytosolic domain (of around 40 kDa) by a single transmembrane helix. The cytosolic domain contains a highly conserved intracellular class III CHD domain (83–87% of sequence identity) followed by a short C-terminal variable region of around 110 amino- acid residues (55–78% of sequence identity) specific to tACs [82]. The presence of multiple putative phosphorylation sites that are conserved in the C-terminal region of the ACs (including the catalytic domain) suggests that this region may be involved in regulation of AC activity [88]. In *T. cruzi*, a quantitative proteomic and phosphoproteomic analysis confirmed that the C-terminal region of an AC isoform upregulated during metacyclogenesis [88] possesses 6 phosphorylation sites [89], which are partially conserved among the different *T. brucei* AC isoforms and may play a role in modulation of AC activity. A similar feature has been described for the kinase homology domain of NPR-A GC [90]. Even though little is known about how ACs are activated (putative natural ligands are still unknown), we do know that activation of these enzymes requires the dimerization of their CHDs [82,91,92]. Typically, this activation occurs under acidic, osmotic (hypotonic) or proteolytic stress conditions (e.g., pH 5.5 or trypsin treatment stimulated AC activity of slender forms, respectively, 10–100-fold and 5–25 fold [83,93]).

Due to the similarity in topology and activation mode between tAC and mammalian receptor-type GC [95] it has been suggested that their extracellular domain may function as receptor [96]. Indeed, similarly to the atrial natriuretic peptide(ANP)-GC receptor, which contains an extracellular Venus flytrap (VFT) domain involved in ligand binding, most of the tACs possess in their N-terminal domain two conserved VFT domains. This may be an indication that these domains are involved in ligand-binding and transport similarly to the prokaryotic extracytoplasmic solute receptors (ESRs) [97] (also called periplasmic binding proteins (PBPs)), which play a role in solute transport systems or initiate chemotaxis by activating flagellar motion. PBPs consist of two globular lobes connected by a hinge region that close around the bound ligand, resembling a Venus flytrap (Figure 4). This modular architecture, repeated in transcriptional regulators such as the *lac* repressors, serves as an extracellular VFT binding module in numerous receptor families. These mammalian receptors include the glutamate/glycine-gated ion channels such as the NMDA receptor, GPCRs, including metabotropic glutamate, GABA-B, calcium sensing, and pheromone receptors, and ANP-GC receptors [98]. Most VFT domains usually form dimers (sensor kinases [99], class C GPCR [100], GC receptor-like [101,102]) that are involved in activation of downstream signaling pathways. Two different and non-exclusive molecular modes of tAC activation were proposed to explain the dimerization through a regulation mechanism promoted by effector binding either to the extracellular N-terminal domain or to the cytosolic region (Δ-subdomain and/or phosphorylation, discussed here). While the regulation through cytosolic region cannot be ruled out, several experiments using analytic gel-filtration chromatography support the possibility that the N-terminal region might regulate activity of the CHD. Whereas a recombinant his-tagged GRESAG4.4B CHD isoform appears to dimerize in vitro and exhibits only low AC activity [91], GRESAG4.1 recombinant CHD isoform only form active dimeric species at low ionic strength, suggesting an involvement of polar interactions that determine a low dimerization tendency at the dimer interface. Moreover, C-terminal addition of a GCN4 leucine zipper to GRESAG4.4B CHD increases the maximal activity by more than 20-fold [91]. This observation argues that both closer proximity of the CHDs in the dimer interface and their relative orientation in this region are fundamental for the regulation of catalytic activity. It also draws attention to the N-terminal receptor-like domain in this regulation (Figure 4) [77]. In addition, receptor-type ACs form dimers in vivo and multimeric complexes are observed under native conditions [94], which suggest that the N-terminal domain may play a role in dimerization.

### 3.2. cAMP Signal Integration in the T. brucei Flagellum, a Complex Organ for Sensing the Environment

Exceptionally for blood-borne protozoan parasites, salivarian trypanosomes are extracellular throughout their life cycle, from the insect vector to the bloodstream of the host, which explains why they have developed several different adaptive strategies as defense mechanisms to enable survival in the two completely different hosts. In the bloodstream of the host *T. brucei* parasites must overcome innate and humoral immune responses, whereas in the insect vector they must resist innate immune responses (meanly oxidative stress, antimicrobial peptides regulated by the immune deficiency (IMD) pathway), and migrate through different compartments of the digestive tract while they undergo several complex developmental changes leading to the final infective metacyclic stage [103]. In these multiple situations, controls for flagellar motility and chemotaxis are fundamental if the parasites are to cope successfully with the different environments they encounter. In bacteria, adaptation to changing environmental conditions is controlled by a two-component system involving a sensor histidine kinase that autophosphorylates in response to a specific stimulus and subsequently transfers the phosphate group to a response regulator modulating its activity, which is usually that of a transcriptional regulator [104]. In many eukaryotic cells, cAMP is a well-known key regulator of flagellar motility and chemotaxis. This is particularly the case of the social amoeba *Dictyostelium*, which is the genetic model of preference for studying chemotactic processes of motility, directional sensing, and polarity [105]. Several indirect observations suggest that the trypanosome flagellum might function in sensory perception and chemotaxis [106]. In a way similar to cAMP microdomains of mammalian cells that participate in the cAMP signal compartmentalization [25], the cAMP signaling in *T. brucei* is highly organized. Indeed most of the elements of the cAMP signaling pathway concentrate in the flagellum of the parasite (Figure 1B), whose membrane lipid composition (high content of sterols, saturated fatty acids, and lipid rafts) differs from other domains of the plasma membrane [107]. Proteomics analysis of *T. brucei* lipid rafts reveals enrichment in many flagellar proteins, such as calcium sensor proteins (calflagins) [107], intraflagellar transport proteins and calcium-dependent cysteine proteases, as well as calpain-related proteins (TbCALP1.1 and TbCALP4.1/CAP5.5) [108]. Interestingly, while TbCALP4.1/CAP5.5 is a classical cytoskeleton-associated protein [109], several other members of the calpains family display discrete differential subcellular localization. An example is TbCALP1.3, an orthologue of TbCALP1.1 that shows a clear enrichment at the flagellar tip [110], suggesting the presence of microdomains in the flagellar membrane. Similarly, different members of insect stage-specific receptor-type AC family, which are upregulated in the insect, localize to similar distinct subdomains of the flagellar membrane. Whereas some AC isoforms are distributed mainly along the entire length of the flagellum, as is the case of the bloodstream-specific ESAG4 [64,66], others are restricted to the flagellar tip, such as ACP1 and ACP4 [94]. These observations emphasize that the flagellar membrane is composed of distinct subdomains, and they support a microdomain model for flagellar cAMP signaling [94]. This distinct localization might be due to a differential glycosylation pattern of these glycosylated ACs [94]. Indeed, a role for glycosylation as a lipid-raft sorting signal has been reported for several lipid raft-associated proteins, such as the mammalian AC8 [111]. In *T. cruzi*, some receptor-type AC isoforms migrate during in vitro metacyclogenesis to the tip of the flagellum, an indication that the tip distribution may be stage-development regulated (S. P. Fragoso and D. Salmon, unpublished results). Thus, it is likely that in trypanosomatids discrete flagellar cAMP gradients generated by different AC isoforms are involved in distinct cellular processes, which must involve the presence of cAMP downstream effectors, AKAPs and PDEs. In *T. brucei* two PDEs were found associated with the parafagellar rod (PFR) structure of the flagellum (TbPDEB2 mostly cytoplasmic and TbPDEB1 linked to PFR [112]) (Figure 1B). In addition, an *in-silico* screen identified several AKAP-like molecules such as the radial spoke proteins (RSP) 3, 4 and 6 [113]. The protein RSP3/AKAP97 [72,114], which is physically linked to the flagellar axoneme, was shown to be required for radial spoke assembly and flagellar motility in *T. brucei* [72]. A PKA-like kinase, which localizes to the flagellum, possesses unique characteristics that differentiate it from its mammalian homologues [35,115]. In place of the N-terminal R2 dimerization domain found in mammals, its regulatory subunit contains an unusual long N-terminus conserved in kinetoplastids that is followed by two tandem copies of a degenerated cyclic nucleotide-binding domain. However, the catalytic subunits of this kinase (encoded by three orthologues) possess all features of a classical PKA in terms of inhibitor and substrate specificity. The unusual structural characteristics of the regulatory subunit might explain the inability of the protein to form homodimers and the absence of detectable activation of the enzyme by cAMP [35]. RNAi-mediated knock-down of the unique gene encoding PKA-R was unexpectedly viable in monomorphic parasites and inhibited forward motility, consistent with a role in flagellar function [116]. The gene encoding PKA-R has been identified in a RNAi screen for stumpy inducers in monomorphic cells (i.e., laboratory-adapted slender cells, that have lost the capacity to generate stumpy forms in vivo). Due to lethality induced by the ablation of PKA-R in pleomorphic cells it was not possible to validate the role of PKA-R in stumpy induction [33].

### 3.3. Intracellular cAMP Function and a Novel cAMP Pathway in T. brucei

The *T. brucei* PKA-like kinase is not activated by cAMP [35], but by cold shock (temperature shift > 10 °C) [115,117]. Instead, several cAMP effectors (CARP1 to CARP4) flagged by Genome-wide RNAi library screening were identified as conferring resistance to the PDE inhibitor CpdA upon knock-down [36,92]. These findings strongly suggest existence of a novel cAMP signaling pathway in these parasites. While CARP1 contains a predicted cyclic-nucleotide binding domain, CARP2 and CARP4 are hypothetical conserved proteins associated with the eukaryotic flagellar proteome [118,119,120], and CARP3 is a hypothetical protein, kinetoplastid specific, such as CARP1. Experimental evidence confirmed that the *T. cruzi* CARP1 orthologue was capable of binding cAMP in vitro, validating the observation that CARP1 may be involved as genuine cAMP effector in *T. brucei* [37]. Although it is clear that cAMP can play a major role in the development of trypanosomes, how it controls or participates in the cell cycle and/or differentiation is presently unclear. Several trypanosomatid differentiation events were thought to be controlled by cAMP [121,122,123,124]. A putative role of cAMP was suggested in the *T. brucei* quorum-sensing pathway, which corresponds to a density-dependent differentiation in bloodstream forms that release an elusive parasite-derived factor (so-called Stumpy inducing factor, SIF) inducing slender to stumpy transformation [125]. Moreover, changes in cellular cAMP levels were observed during the trypanosomatid life-cycle [88,126], such as in bloodstream slender forms where a two to three-fold increase in cellular cAMP level was observed at peak parasitemia, followed by a reduction as stumpy forms took over [121]. In addition, the observation that the cell-permeable cAMP analogue (8-pCPT-2′-O-Me-cAMP) could induce *T. brucei* slender to stumpy differentiation suggested that the cAMP signaling pathway could be involved in this differentiation process [125,127]. However, this conclusion was brought into question by the finding that the active molecules were actually the products of cAMP hydrolysis (adenosine equivalents to cAMP as 8-pCPT-2′-O-Me-adenosine) and not the cAMP itself [127]. While quorum sensing was observed in bloodstream forms, in the insect stage (procyclic form) another social behavior was reported, so-called social motility (SoMo). This intriguing phenomenon, also seen in bacteria, is characterized by the ability of procyclic trypanosomes to aggregate into multicellular groups and to coordinate movement over a solid surface. This is easily observed when parasites are placed on semi-solid agar: they migrate, forming radial projections from a central colony [128,129]. SoMo was restricted to early procyclic forms present in the midgut lumen of the tsetse fly, suggesting its involvement in the migration from the midgut to the ectoperitrophic space [129]. RNAi-mediated knock-down of insect-specific subset of ACs, in particular the dual ablation of ACP1 and ACP2, caused a hyperactive SoMo phenotype [130]. On the contrary, either pharmacological PDE inhibition or RNAi-mediated knock-down of PDEB1, both of, which increased cellular cAMP, blocked SoMo without impairing the viability or motility of individual cells [128,131]. In addition, membrane-permeant cAMP or non-hydrolyzable cAMP analogues have been found to inhibit SoMo, suggesting the direct role of cAMP in SoMo regulation [132]. The observation that a SoMo defect found in PDEB1 knock-downs is restored in *trans* by wild-type parasites argues in favor of a migration factor secreted by wild-type trypanosomes [131]. These data support the idea that ACs and cAMP signaling, possibly involving CARPs, regulate SoMo. A recent study demonstrated that exosomes derived from multivesicular bodies via the endosomal sorting complexes required for transport (ESCRT), which are secreted under certain type of stress such as inhibition of trans-splicing, can affect the migration and possibly participate in the social motility of *T. brucei* procyclic forms [133]. Finally, a mutant strain deficient in N-linked glycosylation was shown to need a greater threshold cell number before migration began, and this mutant appeared to form fewer radial projections on semi-solid substrate than its wild-type parent [134]. Because this mutant infected fewer midguts, it was hypothesized that SoMo might be essential for the parasite to traverse the peritrophic matrix to the ectoperitrophic space. If this turns out to be true, SoMo could be crucial for controlling colonization of the vector in vivo [134].

### 3.4. Role of ESAG4, a Receptor-Type AC Specific for the Bloodstream Form

The expression site-associated gene 4 (ESAG4) represents the prototype gene of the *T. brucei* AC family, and is included in most of the 20–40 polycistronic variable surface glycoprotein (VSG) transcription units [135]. This distribution contrasts with that of the other AC genes termed GRESAG4s (Genes Related to ESAG4), which totalize around 65 copies [65], spread out along the genome [96,136]. Members of the ESAG4 sub-family encode a flagellar AC that is specific for the bloodstream form of *T. brucei* [64] and was the first cyclase to be cloned from salivarian trypanosomes (*T. brucei* and *T. equiperdum*). Its function was identified by complementation of a yeast mutant deleted for AC (*cyr-1*) [64,137].

The mechanism of ESAG4 targeting to the flagellar membrane is still unclear. Although a specific signal (of around 45 amino acids) targeting it to flagellar membrane has been identified in the C-terminal region of several tAC isoforms [94], a deletion mutant that lacked the last 112 residues of ESAG4, including the putative targeting signal, did not affect the flagellar targeting [66]. This finding suggests that flagellar targeting of AC occurs by default while targeting to some subflagellar areas (e.g., flagellar tip) could be signal mediated. As discussed previously, ESAG4 is required for parasite virulence but the intracellular function of this enzyme is still unclear. Several reverse genetics approaches have been used in an attempt to resolve this issue. ESAG4 knock-out from the active expression site did not change total AC activity, because there was a compensatory upregulation of functionally redundant chromosome-internal ESAG4-like GRESAG4 and GRESAG4.1 genes [65]. As a result, there was no effect on parasite growth, either in vitro or in vivo. In contrast, inducible knock-down of the ESAG4 sub-family, including two ESAG4-like GRESAG4s (ACP1-2) that are highly expressed in insect-stage and involved in SoMo (as mentioned above), reduced total AC activity and induced a lethal phenotype linked to impaired cytokinesis [65]. This conundrum was resolved when several conditional ESAG4 DN mutants were generated using a dominant-negative (DN) strategy. This consisted of overexpressing a mutated copy of ESAG4 in which of one of the two metal binding Asp residues (essential for catalysis) and the transition state stabilizing Arg residue (which greatly enhances activity), had been replaced [82]. When overexpressed, these mutants display a transient growth phenotype (over 2 days) due to a cytokinesis block [66] similar to that observed in ESAG4 RNAi cell lines. Phenotype reversion was correlated with up-regulation particularly of GRESAG4.1 gene expression; this up-regulation was also seen in ESAG4 knock-out parasites [65]. Thus, despite being involved in different cellular functions some AC isoforms appear to substitute for others under selective pressure [94]. In addition, the ESAG4 sub-family did not appear to be involved in cellular differentiation from bloodstream form to procyclic form [65]. Instead, the ESAG4 sub-family appears to be required for cell-cycle progression of bloodstream forms. It was proposed that to control cytokinesis ESAG4 sub-family members might be involved in sensing of the VSG coat density [65]. Comparison of total AC activity following hypotonic lysis of two *T. b. rhodesiense* strains, ETat 1.2R (devoid of ESAG4) and ETAT 1.2S (expressing ESAG4), indicated that ESAG4 represents 30–40% of the total AC activity in this parasite (D. Salmon and E. Pays, unpublished data), which is similar to fraction of total AC activity lost upon RNAi-mediated knock-down of the ESAG4 subfamily [65]. The critical level of expression of ESAG4 might be crucial during the transformation event of metacyclic parasites from tsetse fly into bloodstream forms by boosting ESAG4 expression just upon entry into the proliferative phase [65].

### 3.5. Trypanosomal ACs as a Tolerogenic Tool in Mammalian Host Innate Immunity

*T. brucei*, the genetic model of extracellular salivarian parasites, must find a way to deal with both the cellular and humoral immune responses of the host. Like in some pathogenic bacteria (*Anaplasma*, *Borrelia*, and *Neisseria* [138]), chronic infection by these parasites results from antigenic variation involving the expression of a large repertoire of antigenically distinct surface coats, made of densely packaged variant surface glycoproteins (VSGs). To establish a long-term infection and maximize the probability of transmission, the parasites must balance virulence (defined here as proliferation in the host), and pathogenicity (defined here as damage to the host). In mice, control of parasite burden and tissue pathogenicity depends on timely regulation of interactions between two types of population of myeloid cells (M1 and M2-types), which exhibit distinct and opposite activation states. M1-type and M2-type myeloid cells, respectively, refer to cells involved in inflammation (pro-inflammatory) and tissue-healing function (anti-inflammatory). In mice the major site of trypanosome interaction with myeloid cells is the liver [139], where myeloid cells clear more than 80% of parasites [103]. Invading parasites must resist IFN-γ activated macrophage [140] (M1-type myeloid cells) that secretes pro-inflammatory mediators such as type I IFN, TNF-α and NO, and therefore several parasite-derived factors contribute to reduce this response. One of these factors is Kinesin Heavy Chain 1 (TbKHC1), which inhibits NO synthase [141] and another is the cAMP produced by tACs. cAMP inhibits TNF-α secretion by M1-type Ly6C^high^ cells [66] prior to the massive release of immunomodulatory factors (PAMPs) such as the VSG and VSG-derived fragments produced during the first peak of parasitemia [142].

Using a DN version of TNF-α to discriminate between the effects of the membrane-bound (mTNF) versus the soluble form of TNF-α (sTNF), it was recently shown that systemic sTNF has no effect on parasite control or liver pathogenesis. This strongly suggests that it is the mTNF that controls early parasitemia [143]. These results are particularly interesting in light of the mechanism by which the African trypanosome undermines the host’s innate immunity: it uses up to ~50% of its total AC activity to efficiently reduce the host’s capacity to control the parasite in the early steps of the infection [66] (Figure 3). Reduction of parasite control capacity, associated with increased pathogenicity and reduction of host survival, may be a prerequisite for successful infection by low parasite inoculates and/or to ensure a sufficient level of parasitemia for cyclical transmission by the tsetse fly, which is the sole route for parasite dissemination in the field.

The mechanism of tAC activation, which requires the dimerization of the CHD, is still under debate. It could be triggered during parasite phagocytosis into macrophages, by the acidic phagosome environment, or by mTNF-mediated trypanolysis. Only a small number of dead trypanosomes is enough to durably affect host innate immunity [144]. How parasite cAMP can be transferred into host cells remains a mystery. Double transmembrane transfer of cAMP has been reported to occur during macrophage intoxication by *M. tuberculosis* [59] or Treg-mediated immunosuppression (as mentioned above). However, in *T. brucei* another mechanism, one that is related to the highly fusogenic nature of the flagellar plasma membrane may account for the transfer of active tAC into host myeloid cells. Bloodstream parasites can secrete extracellular vesicles (EVs) from nanotubes that originate from the flagellar membrane, and these vesicles appear to contain receptor-type ACs (GRESAG4.4) [145]. Because the formation of EVs is enhanced under stress conditions or by the addition of complement-active FBS [145], synthesis of EVs might be triggered during phagocytic stress. Active AC transfer could occur either by direct fusion of EVs with the host plasma membrane or by phagocytosis (Figure 3).

cAMP-mediated inhibition of TNF-α synthesis through PKA activation in myeloid cells contrasts with TNF-α induction by an AC from intracellular *M. tuberculosis* [59]. Opposing PKA-mediated TNF-α modulation could result from disparate subcellular localization of activated PKA and differential CREB involvement [146]. Indeed CREB was shown to induce transcription of immune-related genes that possess a CRE element, including IL-2, IL-6, IL-10, and TNF-α [42]. Among 17 distinct *M. tuberculosis* ACs, only one (Rv0386) has been shown to be responsible for raising the cAMP level in infected macrophages and increasing TNF-α production via the PKA/CREB pathway [59,69]. In *T. brucei*, most of the 80 ACs appear to be involved in the cAMP burst [66]. This expansion and diversification of the AC family seems to be specific to extracellular salivarian trypanosomes: *T. vivax* (strain Y486) and *T. evansi* (strain STIB805) genomes contain respectively around 30 AC genes (+2 pseudogenes) and 47 AC genes (+10 pseudogenes) (TriTrypDB 35), in contrast to *Leishmania* (around 6 AC genes) and *T. cruzi* (around 15 genes), which are mainly intracellular parasites [66,88]. Why is there such a large repertoire of receptor-type ACs in extracellular salivarian parasites? This polymorphism may be related to the function of AC at the host-parasite interface, which is continuously exposed to the immune system, such is also the case of another extracellular parasite, *Trichomonas vaginalis*, whose genome contains ~123 ACs genes and pseudogenes [147]. Another contribution to the expansion of the AC gene family in salivarian trypanosomes could come from the function of ACs at the vector-parasite interface, owing to the diversity of tissues encountered in the insect vector during the parasite’s developmental cycle [94]. Nevertheless, the relative contribution of the insect-specific AC isoforms to the AC diversity may not be very important if we consider the relatively large number of AC isoforms in *T. vivax*. This parasite has no complex migration within the insect, unlike to the intracellular *Leishmania* parasite, which despite its very low number of AC isoforms, possesses an elaborate cycle in its sand-fly vector. Antigenic diversity due to family expansion of AC extracellular domains might be necessary to prevent efficient recognition by antibodies, as also observed for several parasite enzymes exposed to the immune system, such as *T. cruzi* trans-sialidases [148]. Another explanation might involve a greater ability to change receptor specificity, as shown in *Plasmodium falciparum* where polymorphism in the erythrocyte-binding domain seems to increase parasite fitness [149]. Finally, we cannot exclude that antigenic variation contributes to increase antigenic variability of ESAG4 [65] even if there is no direct evidence that this variability is immunologically significant. Just as a collection of ESAG6/7 copies expressing various host species-specificity of transferrin receptors that may confer an increased capacity of the parasite for adaptation to various mammalian hosts [150], it was suggested that the existence of a battery of slightly different ESAGs (e.g., ESAG4) in the multiple expression sites could provide the parasite with AC isoforms that can regulate growth in response to different environmental conditions [151].

## 4. Concluding Remarks and Perspectives

In trypanosomes, the extraordinary diversity of the AC family, the differential expression and localization of AC isoforms, the evidence for novel downstream cAMP signaling pathways involved in SoMo and the control of host innate immunity highlight both the interest and mysteries regarding these enzymes. Not only are the downstream cAMP pathways still largely unknown, but also the mechanisms of AC activation and the role of the extracellular VFT domains remain to be discovered. Given the unique structural properties and activation modes of the trypanosomal receptor-type AC it becomes clear that these molecules constitute ideal targets for therapeutic approaches. That at least some components of the cAMP pathway of these parasites could be used for drug targeting has already been proposed for TbPDEB1 and TbPDEB2, which possess a parasite-specific hydrophobic P-pocket differentiating them from their mammalian counterparts [152,153]. However, to date there are no drug candidates with sufficiently high selectivity for parasite versus human PDEs that have been found to be safe and effective enough for clinical trials. Therefore, ACs, and in particular ESAG4, might constitute alternatives that can trigger protective immunity against *T. brucei* parasites. Nevertheless, due to the high sequence variability in the N-terminal extracellular domain of ACs, the bet is far from a sure thing. Structural approaches coupled to a high-throughput ligand screening would be necessary to identify in detail the mechanism involved in the activation of these enzymes and discover their Achilles heel. Complete resolution of the cAMP signaling pathway in trypanosomatids will also depend on the technical capacity, still not available, to follow in real time and in single cells the discrete cAMP gradients produced through microdomains of the plasmalemma, in particular at the flagellum. In mammalian cells, cAMP-binding domains of PKA, Epac, or CNG channels-based cAMP sensors have been successfully employed as biosensors to accurately measure the spatially discrete pools of intracellular cAMP in live cells using FRET [154] or patch-clamp techniques [155]. In trypanosomes several attempts for accurately measuring AC activity have been made, but some of these methods present severe limitations in sensitivity [156] and none of them provides an accurate *spatial* localization and temporal signal propagation [131]. New biological techniques such as optogenetics would constitute powerful tools to spatially and temporally control cAMP-dependent signaling in this parasite with the aid of light, as has been done in sperm by using a photoactivated AC that mimics the action of the endogenous AC [157].
